# Anatomy of the Ophthalmic Artery: A Review concerning Its Modern Surgical and Clinical Applications

**DOI:** 10.1155/2015/591961

**Published:** 2015-11-09

**Authors:** Adamantios Michalinos, Sofia Zogana, Evangelos Kotsiomitis, Antonios Mazarakis, Theodore Troupis

**Affiliations:** Department of Anatomy, Faculty of Medicine, National and Kapodistrian University of Athens, Mikras Asias 71 Street, Goudi, 15771 Athens, Greece

## Abstract

Anatomy of ophthalmic artery has been thoroughly studied and reviewed in many anatomical and surgical textbooks and papers. Issues of interest are its intracranial and extracranial course, its branches, its importance for vision, and its interaction with various intracranial pathologies. Improvement of our understanding about pathophysiology of certain diseases like aneurysm formation, central retinal artery occlusion, and retinoblastoma and also invention of new therapeutic modalities like superselective catheterization, intra-arterial fibrinolysis, and intra-arterial chemotherapy necessitate a reappraisal of its anatomy from a clinical point of view. The aim of this review is to examine clinical anatomy of ophthalmic artery and correlate it with new diagnostic and therapeutic applications.

## 1. Introduction

Ophthalmic artery (OA) is the first intracranial branch of internal carotid artery (ICA). It arises soon after ICA emerges from cavernous sinus, follows a short intracranial course, transverses the optic canal, and enters the orbit. There it ramifies in a complex pattern and vascularizes the eyeball and periophthalmic tissues. Most critical branch of OA is central retinal artery (CRA) that vascularizes retina and is of critical importance for vision [1].

According to Bouthillier's classification system, OA is ICA's 5th branch and belongs to its 6th segment. For clinical purposes, ICA is divided into its 1st or cervical segment, 2nd or petrous segment that gives origin to caroticotympanic and vidian artery, 3rd or lacerum segment, 4th or cavernous segment that provides meningohypophyseal and inferolateral trunk, 5th or clinoid segment, 6th or ophthalmic segment that gives origin to OA and superior hypophyseal artery, and 7th or communicating segment where ICA, before its final division, gives origin to anterior choroidal and posterior communicating artery [2].

Pioneering work of Meyer [3] established the so-called “normal” anatomic pattern of OA anatomy. OA was thoroughly studied by Hayreh [4–6] in 1962. Since then many different workers have enriched literature with detailed studies. New therapeutic modalities have redefined the importance of these studies to a major clinical issue.

Knowledge of the detailed anatomy of OA is essential for understanding pathophysiology, diagnostic approach, and therapeutic modalities for its various diseases. Intracranial and intracanalicular course define surgical approach for OA aneurysm management [7, 8]. Microvascularization of optic nerve (ON) head from OA explains clinical image of anterior ischemic neuropathy [9]. OA origin defines proper approach for selective catheterization of OA or superselective catheterization of CRA for treatment of central retinal artery occlusion (CRAO) or retinoblastoma chemoembolization [10].

The aim of this review is to study the anatomy of OA orientated to its clinical applications.

## 2. Material and Methods

An electronic bibliographic search was conducted in Medline Embase, CINAHL, and Cochrane Library for studies on OA anatomy. Terms used were “ophthalmic artery”, “ophthalmic artery aneurysm”, “central retinal artery”, “central retinal artery occlusion”, and “retinoblastoma chemoembolization”. Results were hand-searched and selected appropriately. Moreover, literature of selected articles was further hand-searched for relevant publications. Only articles in English were included in this analysis.

## 3. Ophthalmic Artery Origin

### 3.1. Typical Pattern

OA is the first branch of the ICA immediately after it arises from cavernous sinus and enters the cranial cavity. Its diameter is measured between 0.7 and 1.8 mm [4, 7, 10, 11]. According to Hayreh and Dass [4], in 83.6% of the cases it arises above dura and follows an intradural course, in 6.6% of the cases it arises just above dura, and at 10% it arises below dura and follows a totally or partially extra dural course. Other researchers [7, 12, 13] have published similar results. OA originates from superomedial wall of ICA in 40% of the cases, anteromedial wall in 51%, medial wall in 6%, and superior wall in only 3% of the cases [4]. Those numbers are not universally accepted as other researchers have found different percentages [7, 10, 13].

### 3.2. Variant Patterns

**Figure 1:**
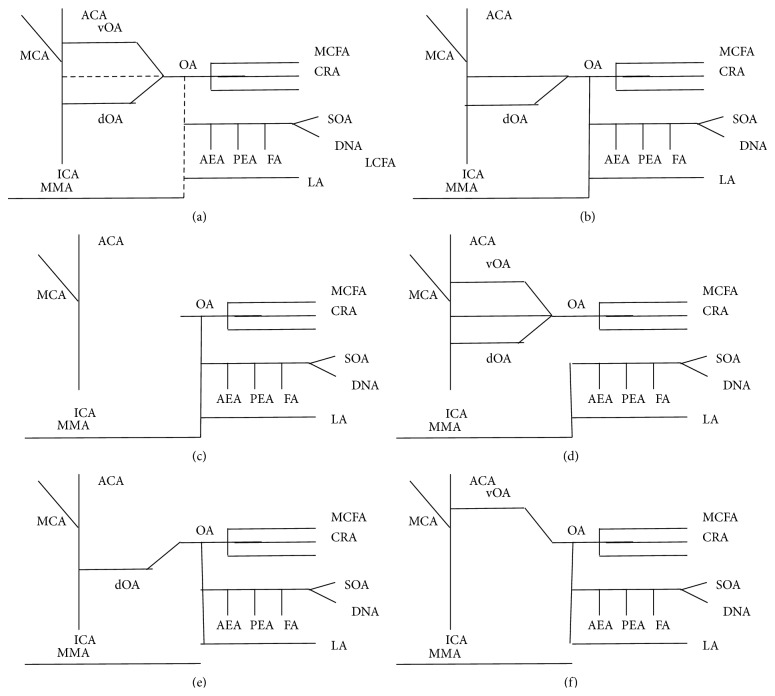
Highly schematic representation of OA's developmental pattern and commonest variations. (a) Overview. Possible anastomoses are represented with dotted lines. (b) Typical anatomical pattern. (c) OA originating from MMA. (d) LA originating from MMA. (e) Persistent dorsal OA. (f) OA originating from ACE, or persistent ventral OA. ICA: internal carotid artery, ACA: anterior cerebral artery, MCA: middle cerebral artery, OA: ophthalmic artery, dOA: dorsal ophthalmic artery, vOA: ventral ophthalmic artery, CRA: central retinal artery, MPCA: middle posterior ciliary artery, LPCA: lateral posterior ciliary artery, AEA: anterior ethmoidal artery, PEA: posterior ethmoidal artery, FA: frontal artery, DNA: dorsal nasal artery, SOA: supraorbital artery, MMA: middle meningeal artery, and LA: lacrimal artery.

For understanding OA variations, a brief review of its embryological development is necessary. In early embryonic life, the eyeball and its contents are supplied by 3 arteries, namely, dorsal OA, ventral OA, and middle meningeal artery (MMA). Those through complex interactions form the adult pattern of OA. At 4–6 mm stage primitive supply derives from “primitive dorsal OA,” a branch of intracavernous ICA that enters the orbit through superior optical fissure. In the same time period, ventral OA appears, a branch of ICA with more cranial origin than adult OA, and enters the orbit through optic foramen. Only later at 16–18 mm stage, those arteries anastomose with the appearance of the stem of adult OA. At this stage, this artery provides vascularization to eye through CRA, lateral posterior ciliary arteries (LPCAs), and medial posterior ciliary arteries (MPCAs). Simultaneously, orbital branches of MMA (then a branch of stapedial artery) enter the orbit through superior optic fissure or a separate canal that might persist at adult life as canal of Hyrtl and supply the orbital contents (but not the eye itself), terminating lacrimal artery (LA). At the end of this stage stem of dorsal OA regresses and the artery itself diminishes as a branch of inferolateral trunk while ventral OA through serial anastomoses and regressions with ICA transfers caudally, at adult OA normal origin. Connection between MMA and OA persists even after translocation of MMA as an External Carotid Artery (ECA) branch at adult life through a developed and clinically important anastomosis between those two systems or as many minute anastomoses. Finally, at 40 mm embryo, adult pattern is established and identified [13–16]. Notably, ECA contribution to the eye diminishes as we ascend evolution tree [5, 14] (Figure 1).

This model explains most cases of variant OA origin through migration, partial/complete regression, and persistence of primitive vessels, and/or remaining anastomotic loops. Commonest variation is OA origin from MMA. Uchino et al. [17] reviewed 1655 magnetic resonance angiographies at a Japanese population and find persistent primitive dorsal OA arising from intracavernous ICA at 0.42% of the cases and OA originating from MMA at 1.45% of the cases. The former can be explained through nonregression of dorsal OA and the latter from regression of both dorsal OA and ventral OA and enhancement of anastomosis between OA stem and MMA. They also found a right-side and male predominance and a correlation between various anomalies of cerebral vessels and OA origin variations. Still this finding can be a diagnostic bias as the authors studied magnetic resonance angiographies of a nonhealthy population.

In rare cases, anastomosis between MMA and internal maxillary artery (IMA) might never develop or regress resulting in MMA originating from OA [18].

Both those variations have significant clinical impact. Persistent primitive dorsal OA complicates dissection for OA aneurysm as it arises intracavernously while OA origin from MMA can lead to blindness in case of accidental or surgical traumatism [10, 13]. Notably in those cases, OA enters the orbit through superior optic fissure rather than optic canal [19]. Even more OA origin from MMA can lead to blindness in case of MMA embolization for head and neck tumors or persistent epistaxis [10]. However, Klufas et al. [20] reported access for retinoblastoma chemoembolization to CRA from MMA if OA catheterization was impossible, thus discovering a catheterization alternative route. According to them chemoembolization for retinoblastoma from MMA can be used solely or supplementary to typical OA catheterization and is an effective, safe, and feasible procedure. Bilateral OA origin from MMA has also been reported [21].

Variant origin of OA from anterior cerebral artery has been reported by Picard et al. [22], Hassler et al. [23], Islak et al. [24], Hannequin et al. [25], and Li et al. [26]. This variation corresponds to the persistence of ventral OA and its main clinical significance is aberrant course of OA above ON, instead of what is presented below. Double OA due to the persistence of both ventral and dorsal primitive OA have been reported rarely [27–30].

Rare anatomic origin of OA from middle cerebral artery [31] and posterior communicating artery [15, 32, 33] has in common agenesis or hypoplasia of internal carotid artery. Finally, an extreme variation, that is, OA origin from basilar artery, has been reported twice and finds no satisfactory explanation [34, 35].

## 4. Course

### 4.1. Intracranial Course and Ophthalmic Aneurysm

After its origin OA follows a short intracranial course until it pierces the dura and enters the optic canal. This distance, although small, (0.5–9.5 mm according to Hayreh and Dass [4]) is of utmost surgical importance as surgical intervention for OA aneurysms takes place at that area. Course of OA between its origin and optic canal is not straight as it creates one or 2 angles. Branches of the OA in the area are deep recurrent OA, an embryologic remnant of dorsal OA that anastomoses with a branch of inferolateral trunk, and superficial recurrent OA that anastomoses with marginal tentorial artery [10].

Aneurysms of the OA cause neurologic symptoms including headaches and diminution of visual acuity. OA aneurysm rupture manifests as subarachnoid hemorrhage with possible fatal consequences. Sometimes OA aneurysms are discovered incidentally during angiography for a different purpose [36]. Some researchers believe that OA aneurysm diagnosis through orbital ultrasound is possible and effective as hemodynamic changes caused by the aneurysm are detectable [37].

Treatment of an OA aneurysm includes embolization or surgical ligation. Surgical access to the area is difficult because of the interference with ON, dural margin of optic canal, and clinoid process. A number of surgical maneuvers like extensive removal of the anterior clinoid process, unroofing of the optic canal, mobilization of the OA, and complete circumferential dissection around the ICA at the level of the dural ring have been proposed for better surgical approach [7, 12]. Nishio et al. [7] suggest a contralateral pterional approach to OA aneurysm. Since OA origin is usually superomedial, contralateral approach allows the neurosurgeon to avoid the obstacle of ON. Visual loss is a significant and often unavoidable complication of procedures for OA aneurysm because of the closure of the ophthalmic artery and manipulation of the nerve or injury to small vessels of OA and retinal ischemia due to temporary occlusions [36].

### 4.2. Intracanalicular Course

After its intracranial course, OA passes below posterior edge of falciform ligament, pierces the dura matter of the ON usually inferiorly, and laterally and alongside with ON enters optic canal. In approximately 6.7% of the cases, origin of OA from ICA is found anterior to falciform ligament, while in very rare cases (<3%) it passes to the orbit separate from ON, in a separate bony canal [4, 12, 14]. The length of the optic canal is small, between 5 and 7 mm [10].

Intracanalicular aneurysms of OA are very rare and have been described only sparsely in the literature [8, 38]. Clinical image is probably more intense due to greater pressure they exercise at ON.

### 4.3. Intraorbital Course and Branches

Intraorbital course of OA divides into 3 parts. OA exits optic canal inferiorly and laterally to ON and courses in close relationship and parallel to it (1st part). Then, it courses medially passing above (83%) or below (17%) to the ON (2nd part). Finally, it is separated to its branches medially to the ON (3rd part). Branching pattern of the OA is very complicated and virtually unique not only between persons but also between eyes of the same person. Depending upon its course superiorly or inferiorly to ON, two different branching patterns can be distinguished [5, 14].

Branches of the OA are CRA, LPCAs, MPCAs, LA, muscular branches, anterior and posterior ethmoidal arteries, palpebral arteries, supraorbital artery and its terminal branches, dorsal nasal artery, and frontal artery.

When OA passes above ON, the first branch is usually a common trunk for MPCA and CRA, second branch is LPCA, 3rd branch is LA, 4th branch is a common trunk for superior rectus and levator, 5th branch is posterior ethmoidal and supraorbital, 6th branch is another MPCA, 7th and 8th branch are muscular branches, 9th branch is anterior ethmoidal, 10th branch is inferior or medial inferior palpebral, and 11th branch is superior palpebral, and then it divides to dorsal nasal and supraorbital artery. Instead when OA passes below ON, 1st branch is LPCA, 2nd branch is CRA, 3rd branch is medial muscular, 4th branch is MPCA, 5th branch is LA, 6th branch is a muscular branch to superior rectus and levator, 7th branch is a common trunk for posterior ethmoidal and supraorbital, 8th branch is a common trunk for superior oblique and medial rectus, 9th branch is anterior ethmoidal, 10th branch is inferior medial palpebral, and 11th branch is superior medial palpebral, and then its final division is to frontal artery and dorsal nasal artery [5, 10, 14, 39].

OA selective embolization has allowed intra-arterial chemotherapy for retinoblastoma, a tumor affecting 1/250.000 children and leading to visual loss metastases and death if left untreated. Standard therapies include systemic chemotherapy and radiotherapy with good results but with serious side-effects, including neutropenia, deafness, metachronous new tumors, and eye enucleation [40]. Intra-arterial chemotherapy for retinoblastoma has demonstrated 2-year eye survival >65% even for advanced cases and >90% for early tumors [40–44]. Safety profile is satisfying, showing only minimal and topical complications like eye inflammation, avascular retinopathy, cataract, and groin hematoma at site of arterial puncture [41, 45].

Intraorbital aneurysms of OA are rare. They usually occur between the 1st and the 2nd parts of OA and they are discovered incidentally either due to exophthalmos or headaches. They do not create visual loss due to ischemia since they are usually located distal to CRA origin [27, 46]. A proposed explanation for their origin is a patent anastomosis between dorsal and ventral primitive OA [47]. Treatment of choice is embolization or surgical ligation. Due to their usually distal location, treatment does not lead to further visual loss [27, 47].

## 5. Branches of Ophthalmic Artery

### 5.1. Central Retinal Artery

CRA is fundamental for vision. It is the first branch of OA in 77.5% of the cases. In 22.1%, it arises from 1st part of intraorbital OA, in 58.7% between 1st and 2nd part and in 18.3% from 2nd part. It arises independently in 37.5%, in a common trunk with MPCA, in 11.5% with LPCA, and in 1.9% in a common trunk with MPCA and LPCA. After a tortuous course, it pierces dura of the ON, usually at its lower and medial part and enters the retina. Rarely (<2%) it can be duplicated. Its length varies between 7 and 20 mm and its diameter between 0.1 and 0.6 mm. After its entry at the retina, it divides into inferior and superior branch which further divide into temporal and nasal branches. Those are visible at fluorescein fundus angiography [6, 9, 10, 14, 39, 48].

CRA is a terminal branch that supplies inner layer of the retina, thus maintaining central role at vision. Central retinal artery occlusion (CRAO) is a devastating disease, responsible for monocular sudden loss of vision with an incidence of 1–8/100.000 people. At 75% of the cases, it is caused by occlusion of CRA by emboli, usually created in carotid arteries or at the heart. The remaining 25% are attributed at atheromatic plaques created in OA itself [39]. Commonest place of occlusion is its narrowest place, where it pierces the dura [39]. An uncommon cause of CRAO is hemorrhage or embolization after accidental entrance at the orbit during endoscopic operations [49, 50].

Ischemia of the retina leads to its necrosis in about 4 h as demonstrated by experiments in monkeys [51]. In a clinical setting, retinal survival time might be longed due to incomplete occlusion of CRA or development of collateral intrachoroid network [52]. An anatomic variation beneficial in CRAO is cilioretinal artery, an arterial branch originating from LPCA or MPCA and supplying part of the retina. Cilioretinal arteries are found in 6–32% of the persons and are bilateral in 14–18%. They usually supply the temporal half of the retina [53]. Furthermore, cilioretinal arteries might create an anastomotic network with normal CRA branches [54] and in rare cases they supply the whole retina [53, 55].

Diagnosis of CRAO is established by clinical history and fluorescein fundus angiography. Emboli are visible and their angiographic appearance is sometimes suggestive of their nature. Small, yellow, and refractile plaques suggest cholesterol emboli, single white plaques suggest calcific emboli, while fibrinoplatelet emboli are seen as small, pale bodies [39]. CRAO has no definitive treatment. Standard treatment modalities include sublingual isosorbide dinitrate, systemic pentoxifylline or inhalation of carbogen hyperbaric oxygen, ocular massage, globe compression, intravenous acetazolamide and mannitol, anterior chamber paracentesis, and methylprednisolone, yet none has shown a better course than natural history of disease [9, 39, 56, 57]. Treatment with intra-arterial fibrinolysis showed promising results in some retrospective studies [58], yet it failed to demonstrate its superiority in a randomized trial and showed worse safety profile [52]. Intra-arterial fibrinolysis might be beneficial and is selected groups of patients, as those with incomplete CRAO [56].

### 5.2. Posterior Ciliary Arteries

Posterior ciliary arteries are branches of the OA supplying the choroid. According to their position, they are termed medial or lateral. Their number varies from 1 to 5, but in 80% of the cases they are 2 or 3. There exists one posterior ciliary artery (always the medial) in 3% of the cases, 2 in 39%, 3 in 48%, four in 8%, and 5 in 2%. Existence of superior or inferior posterior ciliary arteries is inconstant [10, 59].

After their origin, they divide into multiple short arteries that supply proximal choroid and ON head and then pierce the sclera and continue as long ciliary arteries that supply distal choroid, iris, and choroid body [5, 59]. LPCA's diameter is 0.5–0.7 mm as is MPCA's [11]. Posterior ciliary arteries anastomose behind lamina cribrosa and form the circle of Zinn [53].

Posterior ciliary arteries are critical for visual function. They supply directly the choroid and outer layers of the retina. They also participate in inner retina layers supply through cilioretinal arteries and sometimes anastomose with CRA through the circle of Zinn [60]. They also participate in ON hematosis, exclusive at lamina cribrosa, and are alongside with CRA and OA branches at prelaminar and retrolaminar area, respectively. Anterior ischemic optic neuropathy is closely connected to ciliary circulation insufficiency [59]. According to Giuffre [61], posterior ciliary arteries supply the choroid in a segmental manner. Zones between segmentally vascularized areas are amenable to ischemia. Giuffre classified those areas in 6 types and named them “watershed zones.” They identified 6 patterns of watershed zones, namely, Type 1: nasal half of the disc and some choriocapillaris nasal to the disc (3.1%), Type 2a: the optic disc and a band of choriocapillaris nasal to the disc (5.3%), Type 2b: the optic disc and some choriocapillaris along the nasal and temporal edges of the disc (20.2%), Type 2c: the optic disc and a band of choriocapillaris temporal to the disc (21.3%), Type 3: the temporal half of the optic disc and a band of choriocapillaris temporal to the disc (45.4%), and Type 4: an area of choriocapillaris between optic disc and fovea (4.8%). They suspected also the existence of more complex patterns in case of supply by 3 or 4 posterior ciliary arteries. Those were later identified by Hayreh [59].

### 5.3. Lacrimal Artery

LA supplies lacrimal branches and adjacent muscular and periorbital tissues. Embryologically, it originates from MMA. During adult life, this prototype persists in approximately 28% of the cases and thus there is no connection or only minute anastomotic branches between LA and OA. In the rest of the cases, a recurrent branch connects MMA and OA with anastomosis taking place at the apex of superior optical fissure [10]. LA branches are glandular artery (for lacrimal gland), lateral palpebral artery, a small recurrent meningeal branch, and muscular branches [6, 18]. LA diameter is approximately 0.7 mm [11].

### 5.4. Ethmoidal Arteries

There are 2 ethmoidal arteries arising from OA, anterior and posterior. Anterior ethmoidal artery is usually larger than the posterior. It enters the cranial cavity through anterior ethmoidal foramen and becomes anterior meningeal artery. It supplies ethmoidal air cells and the periosteum. Posterior ethmoidal artery supplies superior oblique, the superior and medial recti, and the levator. It also sometimes supplies the nose, ethmoidal air sinuses, and less commonly the falx and dura in the anterior cranial fossa, the periosteum and areolar tissue in the orbit, and very rarely the sphenoid air sinus [5]. The anterior ethmoidal artery diameter is approximately 0.6 mm while the posterior is 0.4 mm [10].

Clinical importance of ethmoidal arteries lies in their role as supplying vessels for meningiomas and vascular malformations. Super selective embolization of OA (beyond CRA origin) can act as treatment or preparation for surgical intervention. In cases, blindness occurs due to retrograde circulation [62–64]. Also ethmoidal arteries can be responsible for epistaxis with their embolization remaining as an effective treatment.

### 5.5. Supraorbital, Frontal, and Dorsal Nasal Arteries

Those arteries have in common exclusive supply to periorbital and extracranial tissues without participating in hematosis of the eyeball or intracranial hematosis. Superior orbital is a small branch that courses at the upper side of the orbit, gives small muscular branches and branches to areolar tissues, and terminates by supplying the scalp. Frontal artery and dorsal nasal arteries are terminal branches of the OA as it exits the orbit. They supply the scalp and the nose, respectively, and their clinical importance lies at their anastomosis with significant ECA branches. Embolization of dorsal nasal branches has been reported for persistent epistaxis [10, 64, 65].

## 6. Ophthalmic Artery Anastomoses

OA has a rich anastomotic network that acts protectively in case of occlusion. In the absence of systemic vascular disease vision remains in 90% of cases of acute proximal occlusion of OA [10]. Anastomotic network divides into deep and superficial.

Deep anastomotic network is formed between IMA and OA. Most important anastomosis is anastomosis formed between MMA and OA, analyzed previously. Other less clinically important anastomoses form between orbital branch of anterior deep temporal artery and LA and infraorbital branch of IMA and inferior palpebral branch of OA [18].

Superficial anastomotic network includes anastomosis of dorsal nasal artery and angular artery of facial artery and anastomosis between superior temporal artery and frontal artery. Superior temporal artery can also anastomose directly with inferior palpebral and lateral palpebral artery through its zygomatomalar and parietofrontal branches. Finally, anastomoses between ethmoidal arteries and alveoantral and sphenopalatine branches of IMA have been recognized which might be responsible for some cases of epistaxis [10, 18, 66].

Clinical importance of communication between ECA system and OA system lies in the possibility of blindness in cases of embolization. Reversely embolization through ECA has been applied in cases of epistaxis with ICA occlusion or chemotherapy delivery through MMA in case of inaccessible OA [65, 66].

## 7. Conclusions

OA's anatomy is complicated. Its dual intracranial and extracranial course, its small size, its close proximity to many significant anatomic elements, especially ON, and its vital importance for vision make it an anatomical and surgical challenge. Improvements in neurosurgery, ophthalmology, and intervening radiology have increased diagnostic and therapeutic options, especially for diseases like retinoblastoma and CRAO. OA also participates in the treatment of intravascular lesion and epistaxis. For those reasons detailed and profound knowledge of its anatomy from a clinical point of view, it is mandatory to ensure the best for the patients' interest.
